# Draft Genome Sequence of *Salmonella enterica* subsp. *diarizonae* Serovar 61:k:1,5,(7) Strain CRJJGF_00165 (Phylum *Gammaproteobacteria*)

**DOI:** 10.1128/genomeA.01322-16

**Published:** 2016-11-23

**Authors:** Sushim K. Gupta, Elizabeth A. McMillan, Charlene R. Jackson, Prerak T. Desai, Steffen Porwollik, Michael McClelland, Lari M. Hiott, Shaheen B. Humayoun, John B. Barrett, Jonathan G. Frye

**Affiliations:** a Bacterial Epidemiology and Antimicrobial Resistance Research Unit, USDA-ARS, Athens, Georgia, USA; b Department of Microbiology, University of Georgia, Athens, Georgia, USA; c Department of Microbiology and Molecular Genetics, University of California Irvine, Irvine, California, USA

## Abstract

Here, we report a 4.78-Mb draft genome sequence of the *Salmonella enterica* subsp. *diarizonae* serovar 61:k:1,5,(7) strain CRJJGF_00165 [also called *S. enterica* subsp. IIIb serovar 61:k:1,5,(7) strain CRJJGF_00165], isolated from ground beef in 2007.

## GENOME ANNOUNCEMENT

The diphasic flagellar antigens of *Salmonella enterica* subsp. *diarizonae* serovar 61:k:1,5,(7) strain CRJJGF_00165 [or *S. enterica* subsp. IIIb serovar 61:k:1,5,(7) strain CRJJGF_00165] distinguishes it from the phylogenetically distinct monophasic *S. enterica* subsp. IIIa serovar 61:k:1,5,(7) (1). *S. enterica* subsp. IIIb serovar 61:k:1,5,(7) is mostly associated with reptiles (2); however, it has been reported in beef, domestic poultry, humans, sheep, and wild birds (3–7). *S. enterica* subsp. IIIb is considered to be of low pathogenicity to humans; however, it has been isolated from ill humans and has the potential to be pathogenic. Here, we announce the whole-genome shotgun data of an *S. enterica* subsp. IIIb strain isolated from ground beef in 2007.

*S. enterica* subsp. IIIb serovar 61:k:1,5,(7) strain CRJJGF_00165 was isolated from ground beef using standard microbiology techniques. SMART-PCR was used to serotype this isolate (8), and the isolate was serotyped with the antigenic formula 61:k:1,5,(7). Susceptibility testing for the strain was performed using broth microdilution plates for the Sensititre semiautomated antimicrobial susceptibility system (TREK Diagnostic Systems, Inc, Westlake, OH, USA), and Clinical and Laboratory Standards Institute (CLSI) guidelines were used to interpret the susceptibility results (9).

Genomic DNA from an overnight culture was isolated using the GenElute bacterial genomic DNA kit (Sigma-Aldrich, St. Louis, MO, USA). DNA libraries were constructed using the Nextera XT DNA preparation kit, and paired-end sequencing was performed on the Illumina HiSeq2500 platform (Illumina Inc., San Diego, CA, USA) using a 500-cycle MiSeq reagent kit. A total of 3,848,000 reads were generated. Reads were *de novo* assembled using Velvet (10) into 108 contigs ≥200 bp with 74-fold average coverage. The combined length of the contigs was 4,782,502 bp with a G+C content of 51.36% and an *N*_50_ value of 119.5 kb. The contigs were ordered with Mauve (11) using the *Salmonella* LT2 genome sequences as references, and coding sequences (CDSs) and tRNAs were predicted with Prodigal (12) and ARAGORN (13), respectively. A total of 4,456 coding sequences (≥50 amino acids) and 53 tRNAs were predicted within the genome. Signal peptides, CRISPR regions, and prophages were predicted using Signalp (14), CRISPRFinder (15), and PHAST (16), respectively. We identified signal peptides in 425 CDSs; one CRISPR locus and no phages were detected in the analyzed contigs. Although this strain was susceptible to all the tested antibiotics, a cryptic aminoglycoside resistance gene, *aac6*-Iy, was detected with ARG-ANNOT (17). The genome data generated for *S. enterica* subsp. IIIb can be helpful to understand why this subspecies does not circulate widely in warm-blooded vertebrate populations, even though it colonizes the intestinal tracks of warm-blooded vertebrates quite well.

### Accession number(s).

The genome sequence of *S. enterica* subsp. IIIb serovar 61:k:1,5,(7) strain CRJJGF_00165 has been deposited in GenBank (NCBI) under the accession number JQYQ00000000. The version described here is the first version.
